# *Post hoc *pattern matching: assigning significance to statistically defined expression patterns in single channel microarray data

**DOI:** 10.1186/1471-2105-8-240

**Published:** 2007-07-05

**Authors:** Randall Hulshizer, Eric M Blalock

**Affiliations:** 1 Department of Molecular and Biomedical Pharmacology, University of Kentucky College of Medicine, Lexington, Kentucky, USA

## Abstract

**Background:**

Researchers using RNA expression microarrays in experimental designs with more than two treatment groups often identify statistically significant genes with ANOVA approaches. However, the ANOVA test does not discriminate which of the multiple treatment groups differ from one another. Thus, *post hoc *tests, such as linear contrasts, template correlations, and pairwise comparisons are used. Linear contrasts and template correlations work extremely well, especially when the researcher has *a priori *information pointing to a particular pattern/template among the different treatment groups. Further, all pairwise comparisons can be used to identify particular, treatment group-dependent patterns of gene expression. However, these approaches are biased by the researcher's assumptions, and some treatment-based patterns may fail to be detected using these approaches. Finally, different patterns may have different probabilities of occurring by chance, importantly influencing researchers' conclusions about a pattern and its constituent genes.

**Results:**

We developed a four step, *post hoc *pattern matching (PPM) algorithm to automate single channel gene expression pattern identification/significance. First, 1-Way Analysis of Variance (ANOVA), coupled with *post hoc *'all pairwise' comparisons are calculated for all genes. Second, for each ANOVA-significant gene, all pairwise contrast results are encoded to create unique pattern ID numbers. The # genes found in each pattern in the data is identified as that pattern's 'actual' frequency. Third, using Monte Carlo simulations, those patterns' frequencies are estimated in random data ('random' gene pattern frequency). Fourth, a Z-score for overrepresentation of the pattern is calculated ('actual' against 'random' gene pattern frequencies). We wrote a Visual Basic program (StatiGen) that automates PPM procedure, constructs an Excel workbook with standardized graphs of overrepresented patterns, and lists of the genes comprising each pattern. The visual basic code, installation files for StatiGen, and sample data are available as supplementary material.

**Conclusion:**

The PPM procedure is designed to augment current microarray analysis procedures by allowing researchers to incorporate all of the information from post hoc tests to establish unique, overarching gene expression patterns in which there is no overlap in gene membership. In our hands, PPM works well for studies using from three to six treatment groups in which the researcher is interested in treatment-related patterns of gene expression. Hardware/software limitations and extreme number of theoretical expression patterns limit utility for larger numbers of treatment groups. Applied to a published microarray experiment, the StatiGen program successfully flagged patterns that had been manually assigned in prior work, and further identified other gene expression patterns that may be of interest. Thus, over a moderate range of treatment groups, PPM appears to work well. It allows researchers to assign statistical probabilities to patterns of gene expression that fit *a priori *expectations/hypotheses, it preserves the data's ability to show the researcher interesting, yet unanticipated gene expression patterns, and assigns the majority of ANOVA-significant genes to non-overlapping patterns.

## Background

In DNA microarray and other massively parallel measurement technologies, analysis of data from two-treatment group experimental designs can be viewed as yielding three 'patterns': 1-significantly upregulated, 2-significantly downregulated, and 3- no significant change. Because the third 'pattern' (no significant change) is typically ignored, only the two patterns, 'upregulated' and 'downregulated', are reported. As more treatment groups are added [e.g., [1,2]], pattern assignment becomes more complex. Although a number of pattern recognition techniques are available [3,4], researchers often choose ANOVA for an overall statistical test.

Faced with identifying/discriminating different patterns of expression among the significant genes, researchers typically employ 'directed' pattern discovery. *A priori *information/assumptions are used to construct templates of expected changes in gene expression across treatment groups [5-10], of which time course based pattern discovery could be considered a specialized subset [11-26]. These approaches are often applied *post hoc *to an overall test. Directed pattern discovery has the advantage of identifying the subset of ANOVA significant results that support the investigator's assumptions. However, disadvantages of this approach include missing unexpected but highly prevalent patterns and not estimating the likelihood of the directed pattern's occurrence by chance. 'Down-weighting' is a unique subset of this directed approach in which the contribution of one or more of the treatment groups is deemphasized [e.g., [1,2]], turning the study into a modified two-group comparison by primarily focusing on differences between only the two 'most important' groups. 'Less important' treatment groups may be used to triage/classify changes between the two important groups, but do not carry equal weight in the overall analysis.

Alternatively, some researchers use 'undirected' pattern discovery approaches, in which patterns of expression are discovered using clustering methodologies, and do not take *a priori *expectations into account [27-30]. These undirected techniques have the advantage of handling highly complex data sets [31,32]. However, estimating the number of clusters is not a trivial process and can dramatically affect the outcome of the analysis [e.g., see [33]] and clusters identified in one study may not be directly relevant to clusters found in another study, although recent advances have been made regarding these determinations [34]. While bootstrapping (e.g., 'Leave-One-Out-Validation') and other techniques can help identify stable clusters [35-37], the likelihood that any given cluster, even a stable one, would have that number of genes by chance can be difficult to assess. Finally, these 'undirected' approaches also can identify important sources of variance that are not associated with treatment. This is a powerful tool for identification of abnormally behaving microarray data and even for the isolation of procedure-related contributions to technical variance, and therefore is critical to microarray analysis and normalization steps. However, this same property can make 'undirected' clustering approaches less desirable for the assessment of treatment-based effects.

All of these approaches are valid and have contributed importantly to microarray-based investigations of biological processes and many array analysis tools have been developed [reviewed in [38]]. Further, new cutting edge techniques merge directed and undirected approaches to allow for more powerful analyses [39]. Finally one of the most highly successful applications (at least in terms of popularity among bench researchers), has been the Significance Analysis of Microarrays (SAM) application [40], which combines multiple testing correction with permutation analysis using classical statistical tests. However, to date no work has been published demonstrating a non-clustering-based approach for treatment-associated, statistically validated gene expression pattern identification within multi-group microarray data.

Here, we developed an algorithm using 1-way ANOVA, followed by all pairwise Fisher's Protected Least Significant Difference (PLSD) testing, to categorize ANOVA significant genes by their expression patterns (as determined by the results of their *post hoc *pairwise comparisons). The number of genes falling into each expression pattern is compared to the number of genes that fall into that pattern by chance (using a Monte Carlo-based random number simulation. The patterns of expression are Z-scored according to their Monte-Carlo-based chance probability estimates. The algorithm was applied to a previously published microarray dataset [2] and discovers patterns reflecting the major findings of that study, as well as a novel pattern with implications for the neurobiology of aging. Further, results from other pattern detection approaches (support tree hierarchical clustering, K-means support with Figures of Merit cluster number estimation, Pavlidis template matching), are compared.

The PPM analysis technique is useful for identifying significant patterns of gene expression within datasets having 3–6 treatment groups that are initially tested by ANOVA. The PPM approach should allow researchers to group significant genes into expression patterns and to estimate probabilities for each of those patterns' occurrence.

## Results

### Algorithm

**Figure 1:**
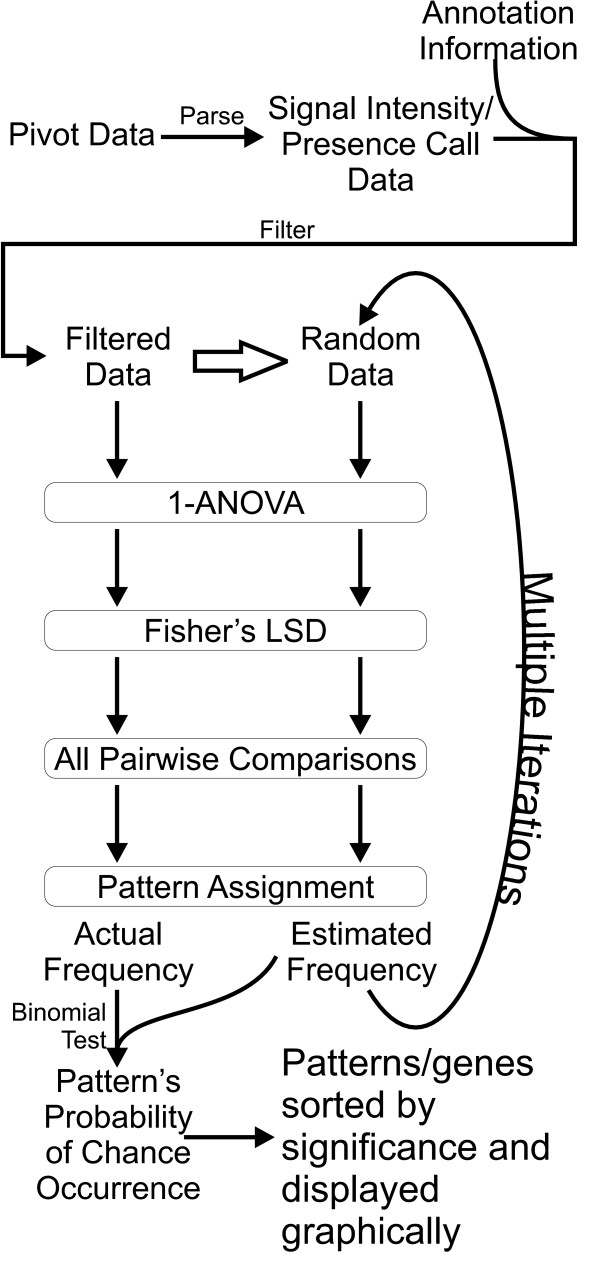
**StatiGen algorithm**. Microarray data (*e.g*., from Affymetrix pivot tables) is parsed into signal intensity and presence/absence calls, and annotation information is appended. Data is then filtered according to user input (*i.e*., absent and unannotated probe sets are removed). Using the Filtered data, StatiGen constructs a Monte Carlo simulation of the data. Both the filtered and Monte Carlo datasets are tested by 1-ANOVA and *post hoc *all pairwise Fisher's PLSD tests. Results from all pairwise comparisons are used to encode pattern IDs (see Methods). Pattern frequency is give by # genes identified in pattern and is statistically compared (Z-test) to that pattern's frequency in a Monte Carlo simulation. Graphic output of significantly overrepresented patterns, along with a list of member genes and annotation information, is included and can be saved as a separate worksheet for further analysis.

Figure 1 depicts the steps involved in the analysis process (StatiGen-specific instructions are included in the software's help file). Steps are discussed in terms of Affymetrix-derived expression array data, although the algorithm is applicable to any data with similar dimensions.

#### Importing

Signal intensity and presence call p-values are often provided by microarray core facilities as either an integrated pivot table, or as separate tables. StatiGen accepts either format and creates two tables, one containing signal intensities and one containing presence call p-values. A third table of annotation information (*e.g*., gene symbols) should also be provided. The first column for these files should contain the same unique identifiers (usually probe_set ID) in the same order (although Statigen provides some limited protection against misaligned data by checking for an equal number of rows in all import sheets, as well as by sorting on the first column).

#### Filtering

The user defines a presence call p-value cutoff (default = 0.05), and then establishes the number of chips that must achieve at least this level of presence (default = 1/2 *n *of the smallest treatment group). We routinely filter out probe sets with no gene symbol annotation [1,2,41,42] as a matter of convenience for subsequent functional grouping analysis. Filtering at this level is also possible (although not required) with StatiGen.

Monte Carlo simulation

A table of random numbers matching the filtered data table's dimensions is created. The random numbers themselves can be regenerated/tested multiple times. Both filtered and random data are run through the following steps (the random data may be run through these steps thousands of times, depending on the iterations selected by the user).

### Gene level statistics

#### Omnibus test

Numerous studies have demonstrated the utility of the Analysis of Variance (ANOVA) approach for microarray studies [43-45]. Here, we apply a basic one way ANOVA (1-ANOVA, see Methods) approach. The mean squared error within (MSE_within_) calculated during the ANOVA is used again in *post hoc *testing.

#### Pairwise comparisons

In the present work, we chose Fisher's Protected Least Significant Difference (PLSD) test. In general, the PLSD test is less conservative than other *post hoc *all-pairwise tests. Therefore, if a significant ANOVA result is found, then Fisher's PLSD is more likely than some other tests to identify a significant pairwise comparison. The p-value cutoff for the the PLSD test is defaulted to 0.05, although users can alter this.

### Pattern level statistics

#### Number of pairwise comparisons

The number of pairwise comparisons is given by the formula '*k *choose 2' as:

*c *= [*k *(*k *- 1)]/2

where *c *is the number of pairwise comparisons and *k *is the number of treatment groups. Thus, a study with 3 treatment groups would have 3 pairwise comparisons, one with 4 treatment groups would have 6 comparisons, one with 5 groups would have 10 comparisons, etc. Each comparison generates three potential results (*r*): 'significant increase', 'significant decrease', and 'not significant'. For each ANOVA-significant gene, the results from all of the pairwise comparisons are encoded into a single 'pattern ID' (see Methods).

#### Pattern ID

We combine results from all of the pairwise comparisons for each probe set, creating a pattern ID. Pattern IDs are constructed using logic gates that use 'increase', 'no significant change', or 'decrease' results from each pairwise comparison. The first pairwise comparison is assigned 1, 0, or -1; the second is assigned 10, 0, or -10; the third is assigned 100, 0, -100 and so on. In this way, the sum of each combination of pairwise comparisons for a given probe set creates a pattern ID encoding that pattern's statistically defined shape, and allowing researchers to easily group different genes that belong to the same pattern. Further, two patterns of opposite sign and the same absolute value will be mirror reflections of one another, which may have value for assessing opposing actions in single pathways [46].

#### Actual and estimated frequencies

Some patterns are statistically more difficult to generate from random data. For instance, patterns in which all pairwise comparisons are significant have a much lower probability of occurring by chance than any other pattern. Therefore, each pattern found in the actual data is assigned its own probability (Z-score) based on that pattern's frequency within the random Monte Carlo simulation (estimated frequency- see Methods).

#### Output

Expression levels for each gene are standardized (so that each gene has a mean of 0 and individual measures are expressed in standard deviations), allowing genes of the same pattern but different signal intensities to be averaged and plotted together. Genes are grouped by pattern and patterns are ranked by overrepresentation significance. Graphs of the mean standardized expression levels for all of the genes in each pattern, along with a list of that pattern's genes, are displayed and can be saved to individual worksheets for further analysis (Fig. 4).

### Limitations

#### Algorithm

**Figure 2:**
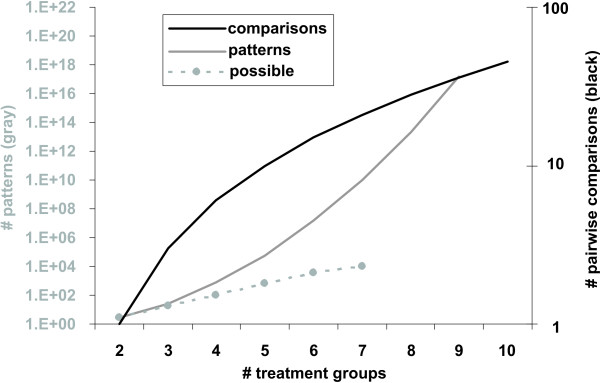
**Complexity increases with number of treatment groups**. Quadratic increase between the number of treatment groups (X axis) and the number of pairwise comparisons (right Y axis) is shown in black. The exponential increase in the number of *post hoc *patterns (left Y axis) is shown in gray. Note that by the time there are seven treatment groups, we predict nearly a billion different patterns. However, due to the lack of independence among the comparisons, the actual number of possible comparisons generated in 100 iterations of a 10,000 gene, 30 array model data system (dotted gray line) is considerably less.

The number of different pattern IDs can be calculated by *PID *= *r*^*c *^where PID is the number of different pattern IDs, *c *is the number of pairwise comparisons and *r *is the number of possible results. The PID value rises exponentially as the number of treatment groups increases (Fig. 2). Because of this, we feel this method is not useful for studies with more than 6 treatment groups, where the number of patterns rivals the number of genes on the chip, obviating the tool's usefulness for reducing complexity. Further, this exponential rise depends on the assumption that the comparisons are independent, when they are actually conditional. Therefore, some patterns (*e.g*., A v B increase, A v C decrease, B v C increase), while predicted by the independent calculation, are not possible in the conditionally dependent data, reducing the number of possible patterns (Fig. 2).

In order to address this issue, only the union of patterns found within the actual data and/or Monte Carlo simulations are tested. This avoids testing for 'impossible patterns'. Presently, we have restricted the test to identification of overrepresented, rather than underrepresented, patterns.

#### Selecting the number of iterations

Exceedingly rare patterns that occur in the real data may not be detected by Monte Carlo. In these cases, the pattern is included as significant, but is flagged. This failure of the Monte Carlo to detect identified patterns is strongly dependent on the number of iterations chosen, the number of treatment groups, and the number of observations within each treatment group. To determine an appropriate number of iterations, we repeat the analysis and observe detected pattern stability. If the pattern detection is stable, then the number of iterations is at least sufficient. If the pattern detection is not stable, then a rule of thumb would be to double the iterations and recheck for stability. The algorithm and software default to one thousand iterations.

#### Excel

**Figure 3:**
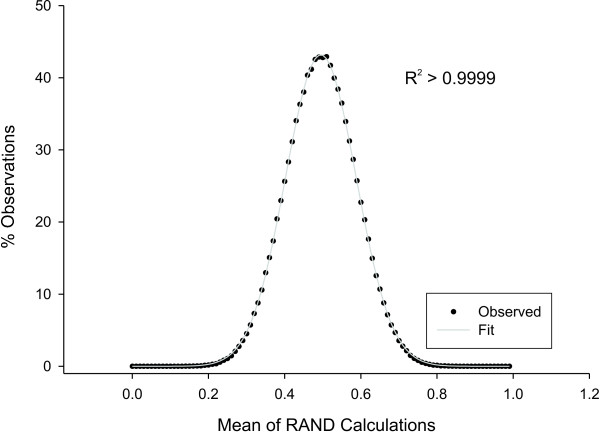
**Normality of Excel's random number generator**. The average of 10 random numbers generated using Excel's RAND function were recalculated one million times, and the % of observations (Y axis) is plotted against the reported mean (X axis). The generated data (black dots) were fit in SigmaPlot (v. 9.0, Systat Software) using a Gaussian model (gray lines- see R^2 ^value in graph).

Statistical calculations in Excel have been reported to be inaccurate in some cases [e.g., [47,48]]. Thus, in the present work, ANOVA calculations were broken down into individual calculations of Total, Within (which was also used for the Fisher's LSD calculation), and Between/Residual sum of squared errors using Excel's DEVSQ function. From these results, F statistics were calculated and the FDIST function was used to look up p-values. These values agree with output in SigmaStat (v. 3.01A, Systat). Finally, the Monte Carlo simulation uses Excel's RAND function, generating evenly distributed values between 0 and 1 (15 decimal places). This does not generate a normal distribution (much like the roll of a single die does not), however, the combined results of multiple RAND calculations do closely approximate a Gaussian distribution (Fig. 3).

### Data reanalysis

#### Blalock et al., 2003 (Gene Expression Omnibus ID: GSE 854)

Nine to ten chips per treatment group, and three treatment groups (Young, Mid-Age, and Aged), were used (see Methods for description). Of the 8799 probe sets, 5865 were rated present (having 5 or more chips with 'present' calls) and 673 were significant by 1-ANOVA (p < 0.05). Of these ANOVA significant probe sets, 2 probe sets did not have any significant *post hoc *Fisher's PLSD comparisons, 138 were significant between Mid-Age and Age; 353 between Young and Mid-Age; and 497 between Young and Aged. A Venn diagram (Fig. 5) shows the relative overlap among the three pairwise comparisons. Nearly three quarters of all genes found significant by ANOVA were also significant by the Young vs. Aged comparison. The Young vs. Mid-Age comparison was the second strongest comparison and Mid-Age vs. Aged had the fewest significant comparisons (statistically, each of these pairwise comparisons have the same probability of identifying genes).

**Figure 4:**
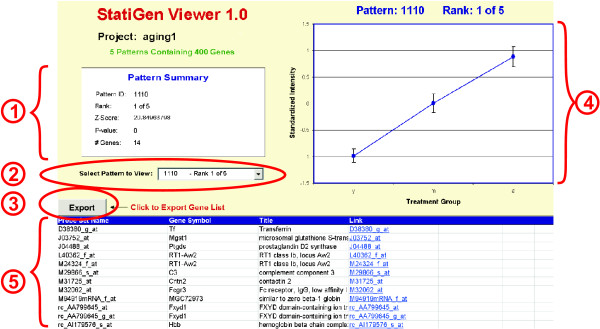
**StatiGen output example showing a significantly overrepresented pattern**. **1**. The pattern summary box gives details regarding the pattern's significance, rank among significant patterns, and # genes in pattern. **2**. Below the pattern summary box, there is a drop down menu allowing users to rapidly switch their view to other significant patterns. **3**. The export button allows users to export their list to another Excel worksheet for further analysis. **4**. A graph of the average (± SEM- standard error of the mean) of the standardized expression values from all genes in the pattern (Y axis) across the treatment groups in the study (X axis) is displayed. **5**. The list of this pattern's member genes is presented. The first column is probe set ID, and the last column is hyperlinked to the National Cancer Institute's database of Affymetrix probe set IDs. The intermediate columns are provided by the user at the 'annotation information' stage of the algorithm (see Fig. 1).

**Figure 5:**
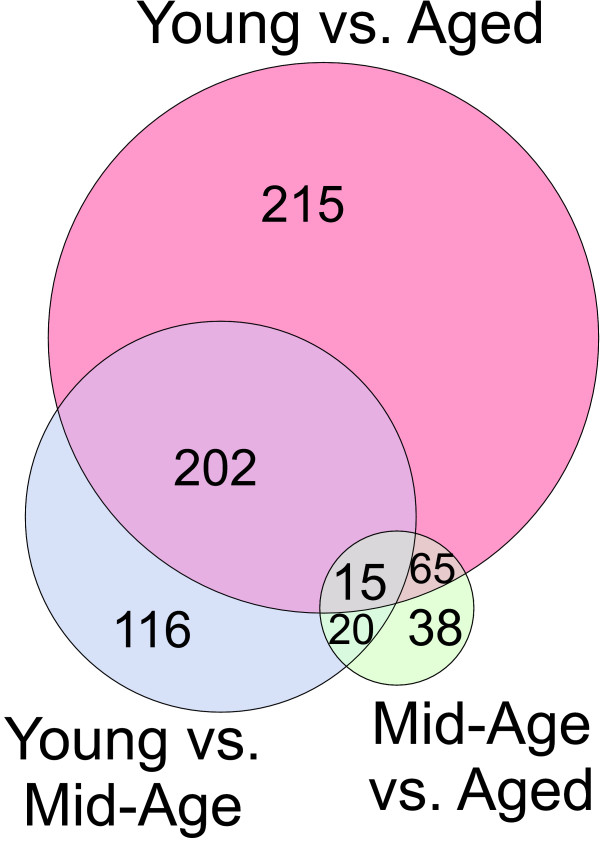
**Venn diagram of pairwise comparisons**. Among ANOVA significant (p ≤ 0.05) genes from the Blalock et al., 2003 study, all possible pairwise comparisons were applied using Fisher's PLSD. The Venn diagram shows the number of ANOVA significant genes that were significant in at least one pairwise comparison, and notes overlap (direction of change was ignored). Interestingly, although all pairwise comparisons had an equal probability of detecting genes, the Young vs. Aged comparison was clearly the strongest comparison, and Mid-Aged vs. Aged was clearly the weakest.

**Figure 6:**
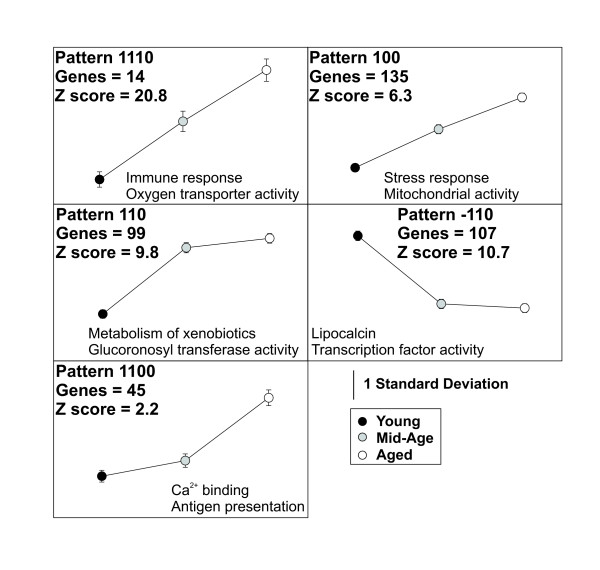
**Significantly overrepresented patterns**. Five significantly overrepresented patterns are plotted, along with highlighted functional categories found to be overrepresented in each pattern (complete lists of each pattern's probe sets in Additional file 3).

When pairwise comparisons are considered in concert using StatiGen's *post hoc *pattern matching algorithm (Fig. 1), interesting patterns emerge. Five of twenty-four patterns are significantly overrepresented (Fig. 6 and Table 1). The list of significant genes contained within each pattern (Additional File 1) was uploaded to: 1) DAVID [49] website and compared with a custom background list containing all probe sets in the study rated present and annotated, and 2) Onto Express [50] and contrasted with the RG-U34A chip as a background. Some selected functional categories that agreed between the two analyses and appeared to represent biological processes of the individual patterns are listed (Fig. 6).

**Table 1:** PPM-Defined Expression Patterns

**Pattern ID**	**# Found**	**# Expected**	**Z-Score**
1110	14	0	20.84964
-110	107	40	10.67321
110	99	38	9.826184
100	135	81	6.297468
1100	45	32	2.15279
-1110	1	0	1.089534
-100	83	80	0.306635

*Pattern ID*: Unique pattern number assigned by StatiGen to combination of pairwise comparison results. *# Found*: # Genes in the actual data identified as members of that expression pattern. *# Expected*: The number of genes one would expect based on the Monte Carlo estimation of chance occurrence. *Z score*: The distance (in standard deviations) away from the chance occurrence probability. Note that StatiGen does not report underrepresented patterns (negative Z scores; see Results [limitations]).

The most significant pattern (1110) was not the pattern with the most genes, but one among six possible patterns with the least likelihood of occurring by chance (all three pairwise comparisons significant). Many of the genes in this pattern reflect a well-characterized and robust increase in inflammatory markers seen in our and other researchers' microarray-based studies of the aging brain [51-53]. Also note that pattern 100 reflects a weaker, but significant monotonic rise with aging that appears to contain genes associated with similar functional categories.

The second and third most prevalent patterns are mirror reflections of one another (110 and -110), and highlight genes whose expression levels were significantly different in two comparisons (Young vs. Aged, and Young vs. Mid-Aged), but no different in the third (Mid-Aged vs. Aged). Downregulated in Aged relative to Young (-110) genes in this category are enriched in immediate early genes (*e.g*., transcription factor activity) and genes associated with intracellular signaling cascades (*e.g*., lipocalcin). Upregulated in Aged relative to Young (110 pattern) genes included functional categories associated with stress response (*e.g*., Metabolism of Xenobiotics and Glucoronosyl transferase activity).

Finally, although there were relatively few genes that were significantly changed from Mid-Age to Aged, a subset of genes (pattern 1100: no significant difference from Young to Mid-Age, a significant difference from Young to Aged and from Mid-Age to Aged) was rated as significantly overrepresented by StatiGen and included genes related to calcium binding and antigen presentation.

### Comparison to other approaches

The PPM algorithm was developed to assign statistical probabilities to patterns identified *post hoc *to 'per gene' statistical testing in a multi-treatment group setting, and shares some features with other approaches. Therefore, in this section we compare PPM output to two popular clustering approaches that use resampling techniques to assess stability (Support Trees and K-Means Support), as well as a template matching approach (Paul Pavlidis' Template Matching, PTM; [6]), using TIGR's MeV software [54]. Standardized gene expression data for the probe sets previously identified as present and annotated were imported into MeV. Because the goal of StatiGen is identification of patterns present among the ANOVA-significant (and therefore heavily treatment-group biased) data, these other approaches were also applied to the ANOVA-significant genes.

#### Support Trees (Figure 7)

**Figure 7:**
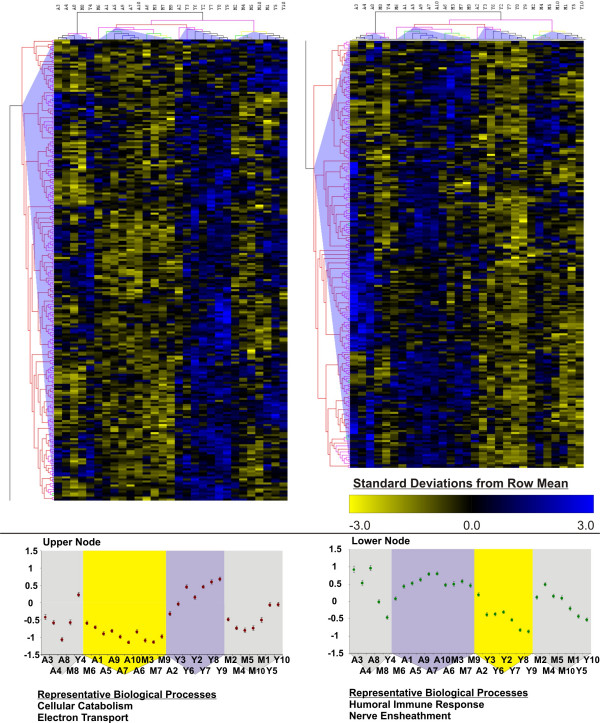
**Support Trees**. Hierarchical clustering using MeV software with 100 bootstrap iterations on samples and experiments was used to generate a dendrogram. For display purposes the most stable Upper Node (left) and Lower Node (right) are separated for display purposes. Branches in the dendrogram are color-coded according to reliability (as a percentage of all bootstrap iterations in which cluster was identified: Black- 100%, Gray 90–100%, Blue- 80–90%, Green- 70–80%, Yellow- 60–70%, Orange- 50–60%, Fuscia- 0–50%, Red- 0% and Pink- unrecovered), and shaded triangular areas are used to consolidate subjects/genes for display. Beneath each section of the dendrogram is a graph depicting mean standardized intensities for highlighted groups of subjects that were reliably clustered with one another. Below the graphs are representative Gene Ontology over-expressed Biological Processes for Upper and Lower nodes. Gene expression intensities are expressed in standard deviations from the mean for each gene (see scale bar).

Support Trees is a version of hierarchical clustering that uses bootstrap methods to establish branch stability. Here, we used Pearson correlation as a distance metric, average linkage as a linkage method, and clustered on both genes and experiments using one hundred bootstrapping iterations. Branches are color-coded according to stability (see caption text). Genes could be reliably divided into two groups (left and right panels) but showed highly unstable branching patterns at lower levels (mainly red- 0% support) while experiments were more stable, with a majority of aged chips being separated from their young and middle-aged counterparts. Further, subsets of chips formed highly stable experimental clusters (from left to right): [A3, A4, A8, M8, Y4- a mix of different age groups], [M6, A1, A5, A9, A7, A10, A6, M3, M7, M9- the majority of aged subjects], [A2, Y3, Y6, Y2, Y7, Y8, Y9- the majority of Young subjects] and [M2, M4, M5, M10, M1, Y5, Y10- the majority of Mid-Aged subjects]. Finally, it appears that the two experimental clusters most specifically enriched in aged vs young subjects (the middle two experimental clusters), in large part drove the discrimination of the genes into the left and right panels, with the outer two experimental clusters contributing relatively little information at this level of branching.

#### K-Means Support (KMS; Figure 8)

**Figure 8:**
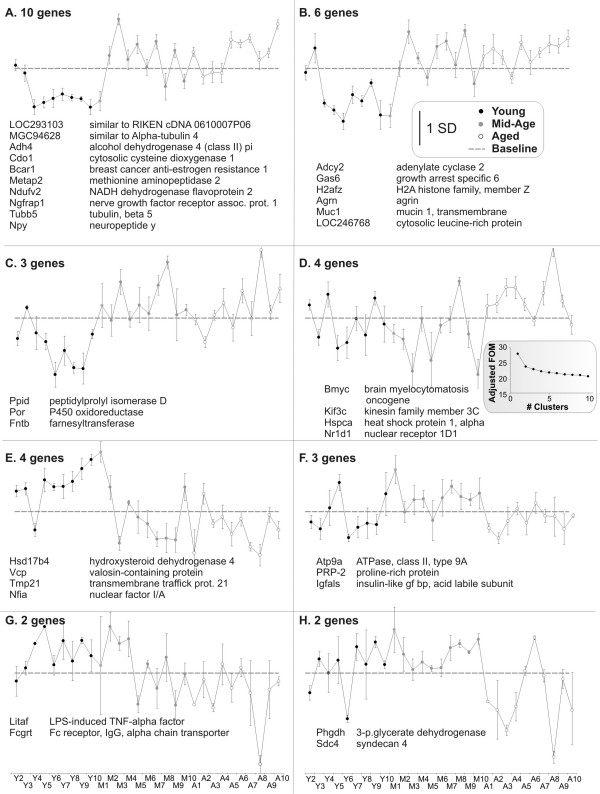
**K-Means Support (KMS)**. The KMS algorithm with 100 iterations and Pearson correlation for distance, established eight 'consensus clusters' that appeared in at least 80 iterations. For each panel, individual observations are plotted along the X-axis and standardized signal intensity averaged for genes in the cluster, is plotted along the Y-axis. For reference, a '0 line' has been added indicating baseline (scale bar = one standard deviation). Within each panel, the members of the cluster are indicated. **Panels A, B, E**) These clusters show an apparent discrimination between young subjects and all others. **Panel C**) The cluster most analogous to a monotonic increase. **Panels D & F-H**) These clusters appear to be isolating patterns based on non-treatment effects. **Inset**: Figure of Merit (FOM) procedures were used to estimate number of clusters for K-means but results were difficult to interpret.

KMS uses the K-Means clustering algorithm run multiple times (here, 100 times) to establish 'consensus clusters' that appear in at least 80% of the iterations, again demonstrating clustering stability. The Pearson correlation metric was used for distance, and Figure of Merit (FOM) procedures were used to estimate number of clusters for K-means. FOM analysis was difficult to interpret, showing that more than one cluster was present, but indicating a relatively flat line effect out to 20 clusters (graph in inset truncated at 10 clusters). Using a combination of information from FOM, and previous analyses by StatiGen, we selected five clusters as a starting point for KMS. In the resulting procedure (Fig. 8) KMS ran 100 five-cluster iterations and reserved the genes that were clustered together in at least 80 of those iterations. The resultant set of genes fell into eight clusters which are depicted in Figure 8. However the majority of ANOVA-significant genes (93%) failed to be assigned to a cluster.

**Figure 9:**
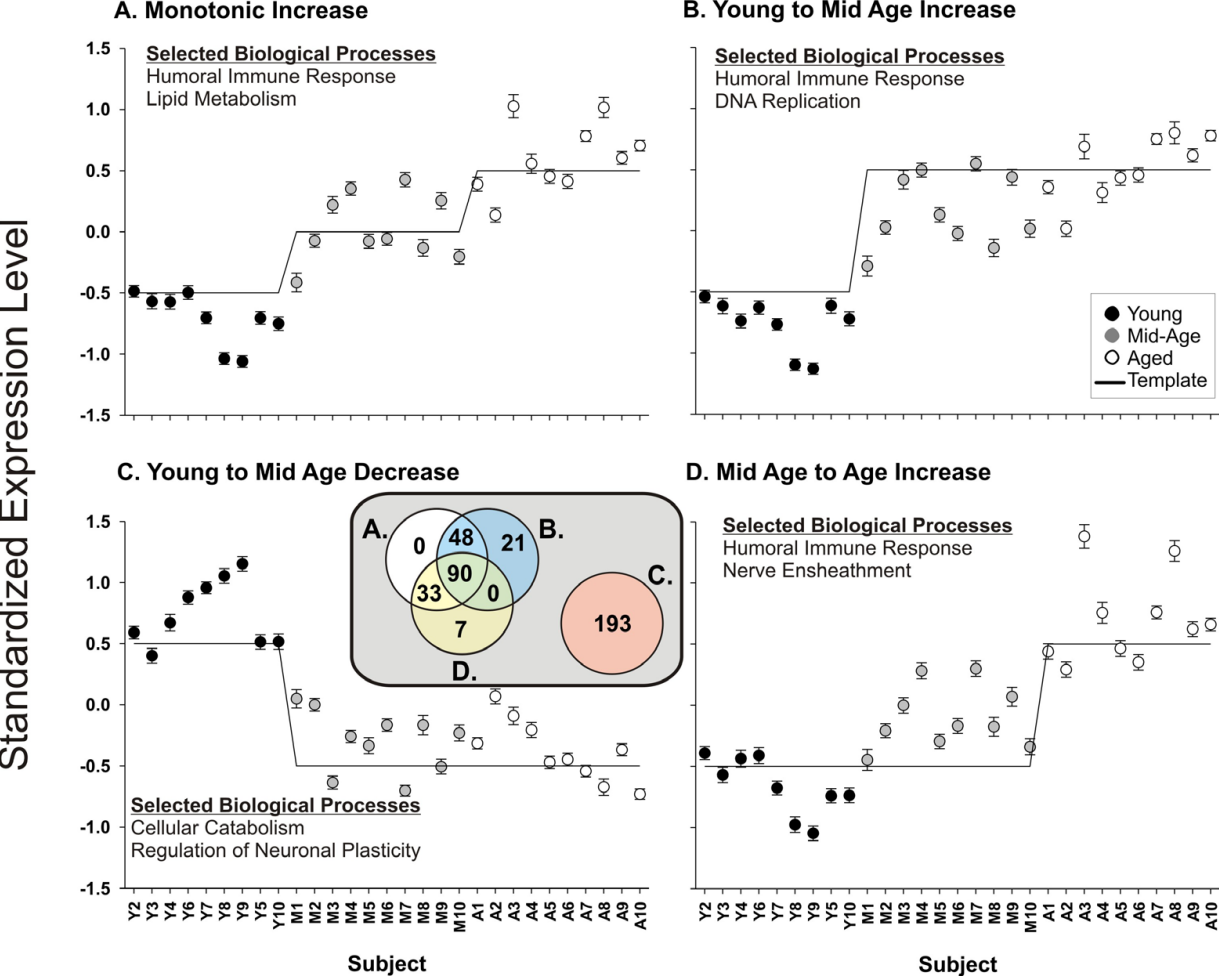
**Pavlidis Template Matching (PTM)**. Four PTM templates were created. For each template, the standardized signal intensity average of all genes that fit the template are plotted by subject along with a superimposed template to which they were correlated. **A**. The 'monotonic increase' template matches two StatiGen-identified patterns, 1100 and 100 (Fig. 6). **B**. Young to Mid-Age increase corresponds to pattern 110 (Fig. 6). **C**. Young to Mid-Age decrease corresponds to pattern -110 (Fig. 6)- the mirror reflection of pattern 110. **D**. Mid-Age to Age increase corresponds to pattern 1100 (Fig. 6). Genes in each pattern were tested for overrepresentation of Biological Processes in the Gene Ontology using DAVID (see Methods). Selected overrepresented categories are listed in each panel. **Inset**: High degree of overlap between A, B, and D, with the three templates identifying similar sets of genes (by overrepresentation analysis, humoral immune response genes could not be distinguished among the three templates).

**Pavlidis Template Matching (PTM; Figure **9**)**

The PTM approach allows researchers to construct a 'template' expression pattern and use Pearson's correlation to identify genes that significantly correlate with that template. Here, we chose to apply PTM to those genes that were significant by ANOVA, effectively turning the PTM procedure into a *post hoc *test. PTM investigates one user-defined pattern at a time and we used StatiGen-identified patterns to establish templates for PTM.

The two monotonic patterns found by StatiGen (Fig. 6, patterns 1110 and 100) were fit by the same template in PTM (Fig. 9A), and, among the age-upregulated patterns identified by StatiGen, this monotonic increase template found the largest number of genes in PTM. However, other upregulated patterns (Panels B and D) also found a large number of highly overlapping genes in PTM as evidenced in the Venn diagram (inset), as well as the highly analogous Biological Processes found to be overrepresented among genes identified panels A, B, and D. Decreased expression patterns from Young to Mid-Age, and sustained through Age using PTM (Fig. 9C) revealed a completely non-overlapping set of genes that were related to cellular catabolism and neuronal plasticity- supporting previous work suggesting that neuronal involution may play a critical role in cognitive deficits seen with aging.

## Discussion

### Prestatistical filtering

Researchers often triage microarray results with metrics that are blind to treatment groups, such as spot quality, signal intensity, and/or Microarray Suite 4 or 5 (MAS4 or MAS5) derived 'presence' calls. Such approaches can dramatically improve statistical performance and reduce the error associated with multiple testing [e.g., [55,56]]. There are a number of methods for such filtering. Observations that fall below some criterion could be treated as missing values, artificially brought up to a minimum intensity value, weighted according to the strength of the quality control measure, or the number of chips for which a given probe set exceeds some threshold value can be calculated. In the present work, we have opted for the latter approach.

### Statistical tests

A number of different approaches could be used to assign significant results to the data (*e.g*., fold change, coefficient of variance, volcano plot style combinations of p-values and fold changes). In theory, any of these approaches would work as an initial step for the detection of patterns within the data. In the present work, we focus on predicted reliability, rather than magnitude, of change.

Interestingly, studies in which magnitude of change, irrespective of variance, are applied (*i.e*., fold change) require an *a priori *assumption on the part of the investigator, that some level of change is necessary for a biological effect to be exerted, and further, that such a level of change is the same across all expressed genes. Moving to statistical criteria ignores potential biological effects, instead focusing on the degree of variance and the likelihood that such a difference in means, given the variance of the measures, could have occurred by chance. Thus, the statistical results infer relative security of findings, but it is still up to the investigator to ascertain the biological meaning (or lack thereof) of any change. A change in gene X may be very reliable, yet epiphenomenal with regard to the biological process under investigation.

Other pairwise comparisons would be appropriate *post hoc *to the ANOVA (*e.g*., Scheffe's, Tukey's). In the present work, we chose Fisher's Protected Least Significant Difference (PLSD) test. In general, the PLSD test is less conservative than other tests. Therefore, if a significant ANOVA result is found, then Fisher's PLSD is more likely than some other tests to identify at least one significant pairwise comparison. By assembling genes into their *post hoc *defined patterns, the statistical reliability of the pattern may 'protect' statistically weaker findings. This approach has been used to great effect in functional grouping analysis of microarray data [see [41,50,57-59]].

### Patterns found in Blalock et al., 2003

The PPM method applied with StatiGen confirmed and extended the work of the original paper, finding that a majority of genes had changed by Mid-Age, and identifying upregulated inflammatory genes and downregulated genes related to neuronal function. The number of genes significant by each pairwise comparison (Fig. 5) alone is often useful, helping researchers determine which comparisons show the largest number of significant results (suggesting treatments with the most powerful effects on the transcriptome). Here, the expected result, that the greatest age-dependent difference in transcriptional profile would be between the Young and the Aged groups, was clearly supported by this analysis. However, such approaches are limited in their ability to assess a particular comparison's effects on the transcriptome while simultaneously appreciating the effects of other comparisons.

Further, StatiGen identified a significantly overrepresented pattern associated with a selective, Mid-Age to Aged change, and many of the genes in this pattern are associated with calcium dysregulation, a well-supported hypothesis of neuronal dysfunction and cognitive deficit in aging [60-62]. Thus, this approach identified not only age-related and possibly precipitating causes of age-related cognitive deficits in an animal model, but was also able to isolate a pattern of expression that directly and temporally correlated with that cognitive decline.

Four of the five identified patterns (1110, 100, 110, and -110) strongly validate conclusions of the original study [2] that transcriptional levels in the Mid-Age group are generally intermediate between Young and Aged groups, or are similar to the Aged group. Further, the Mid-Age animals, although they had yet to show a statistically significant cognitive deficit, generally had transcriptional profiles more similar to Aged than to Young hippocampal CA1 regions. The identification of these patterns by StatiGen highlights the unambiguous manner in which patterns can be defined and examined, and further highlights, at least in the example shown, that the conclusions of the researchers regarding transcriptional changes were largely supported by the data.

The genes comprising the fifth pattern (1100) may be of particular interest as their expression levels inversely correlate with behavioral deficits observed with age (a moderate and non-significant decrease by Mid-Age, followed by a significant drop-off in the Aged group). Interestingly, many of the genes found here represent inflammatory (e.g., Lps, S100 A1 and A9, Rt1Dmb) and astrocyte/oligodendrocyte processes (e.g., Gfap, Mobp, Mag), suggesting that these potential biomarkers may influence, or be influenced by, cognitive status changes with age.

The potential interactions among oligodendrocytic, myelin, and inflammation related genes, were a key, novel proposition in the original work. The finding here supports that interaction's potential role in cognitive deficits with age. Importantly, perturbed calcium homeostasis seen here has been a long-standing hypothesis of brain aging [reviewed in [60-63]] supported by numerous studies [e.g., [64-67]]. In the present context, it suggests that calcium signaling perturbations are common to many cell types in the brain. Further, altered calcium and inflammatory changes together suggest that other popular aging hypotheses [e.g., reactive oxygen species, see [68,69]] may all play a role in altered cognition with aging. This pattern's discovery therefore highlights the PPM algorithm's second strength, discovery of patterns that were not anticipated (based on the results of the previous work).

### Other methods

As expected, support trees applied *post hoc *to the ANOVA showed a strong tendency to group subjects according to treatment, as the ANOVA selection should heavily bias this procedure towards treatment-based clustering. However, expression pattern identification among genes was not as refined, with a relatively stable discrimination between up and down regulated genes among two of the four experimental clusters, and other patterns of expression showing poor replication.

K-means support, in conjunction with Figure of Merit estimation of cluster number, reliably identified eight clusters but was unable to assign more than 90% of the ANOVA significant genes. This suggests that some KMS parameters may need further adjustment, the data may need further transformation, the ANOVA criterion is inappropriate, or that this approach is not adequate for this data set.

Pavlidis template matching (PTM) clearly identified sets of genes using statistical Pearson's correlation probabilities. However, because each fitted template is performed in isolation, there is a high degree of overlap between different, but related patterns of expression. One way to reduce the degree of overlap would be to increase the p-value stringency criterion for inclusion in each template. However, increased stringency would also reduce the proportion of the ANOVA-significant data set identified by the procedure. Interestingly, the PTM approach does point to a potential improvement of the PPM strategy employed by StatiGen. Presently, the PPM procedure considers each unique combination of pairwise contrast results as a separate pattern. However, it is possible that, like the PTM procedure, two patterns that completely correlate with one another in PPM (e.g. Fig. 6, patterns 1110 and 100) could be merged, reducing the complexity of pattern output in PPM.

## Conclusion

The PPM algorithm was born of necessity in our microarray research dealing with multiple group studies and the relatively large amount of data generated using arrays [70]. Although newer methodologies are greatly improving undirected approaches at both the gene expression and functional analyses levels ([71-73], cluster number estimation, statistical likelihood of a cluster's occurrence, and gene membership across iterations are still important issues. Directed approaches are unable to detect unexpected patterns, as the discriminating features of the patterns must be determined *a priori *by the investigator. The PPM algorithm's implementation in StatiGen skirts these issues: pattern number and statistical likelihood are defined and estimated, and gene-to-pattern assignments are stable. However, these improvements come at the cost of limited complexity reduction. Large numbers of treatment groups (e.g., > 6) are inadequately handled by this process because the number of patterns increases with increasing number of treatment groups (Fig. 2).

Applied to a published microarray experiment, the StatiGen program successfully flags patterns that had been manually assigned in prior work, and further identifies other gene expression patterns that may be of interest. Thus, over a moderate range of treatment groups, PPM appears to work well, allowing researchers to assign statistical probabilities to patterns of gene expression that fit *a priori *expectations/hypotheses while still preserving the data's ability to show the researcher interesting, yet unanticipated gene expression patterns.

Important future work with this approach will include adding the option to identify and merge highly similar patterns, convert the software language to R format, and provide options for noise reduction/outlier removal prior to analysis.

## Methods

### PPM algorithm

The *post hoc *pattern matching algorithm was created stepwise in Excel (v. 2003, SP2, Microsoft). All statistical calculations were verified in SigmaStat (v. 3.0, SyStat) on representative probe sets.

### Statistics

Here, we apply a basic one way ANOVA approach, where each probe set is tested individually, and total sum of squared variance is partitioned into variance attributable to treatment, and the remainder is considered residual.

∑i,j(yij−y¯)2=∑i,j(yij−yi¯)2+∑i,j(y¯i−y¯)2
 MathType@MTEF@5@5@+=feaafiart1ev1aaatCvAUfKttLearuWrP9MDH5MBPbIqV92AaeXatLxBI9gBaebbnrfifHhDYfgasaacH8akY=wiFfYdH8Gipec8Eeeu0xXdbba9frFj0=OqFfea0dXdd9vqai=hGuQ8kuc9pgc9s8qqaq=dirpe0xb9q8qiLsFr0=vr0=vr0dc8meaabaqaciaacaGaaeqabaqabeGadaaakeaadaaeqbqaamaabmaabaGaemyEaK3aaSbaaSqaaiabdMgaPjabdQgaQbqabaGccqGHsisldaqdaaqaaiabdMha5baaaiaawIcacaGLPaaaaSqaaiabdMgaPjabcYcaSiabdQgaQbqab0GaeyyeIuoakmaaCaaaleqabaGaeGOmaidaaOGaeyypa0ZaaabuaeaadaqadaqaaiabdMha5naaBaaaleaacqWGPbqAcqWGQbGAaeqaaOGaeyOeI0Yaa0aaaeaacqWG5bqEdaWgaaWcbaGaemyAaKgabeaaaaaakiaawIcacaGLPaaaaSqaaiabdMgaPjabcYcaSiabdQgaQbqab0GaeyyeIuoakmaaCaaaleqabaGaeGOmaidaaOGaey4kaSYaaabuaeaadaqadaqaamaanaaabaGaemyEaKhaamaaBaaaleaacqWGPbqAaeqaaOGaeyOeI0Yaa0aaaeaacqWG5bqEaaaacaGLOaGaayzkaaaaleaacqWGPbqAcqGGSaalcqWGQbGAaeqaniabggHiLdGcdaahaaWcbeqaaiabikdaYaaaaaa@5CDA@

where *y *is the observation, *i *is one of *k *groups, and *j *is the number of observations within group. 'Between sum of squared error' degrees of freedom equals the number of treatment groups -1 (*k *- 1) and 'Within sum of squared error' degrees of freedom equals the total number of observations – the number of groups (*N *- *k*). The summed errors are divided by their respective degrees of freedom to produce their mean squared errors. A ratio of between/within mean squared error generates the F-statistic, which, along with the degrees of freedom for the numerator and denominator of the F-statistic (*k *- 1 and *N *- *k*, respectively), is used to generate a p-value for each probe set. The mean squared error within (MSE_within_) is used again in *post hoc *testing.

*Post hoc *to a significant ANOVA, Fisher's PLSD follows the form:

LSD=2•MSEwithin•F1,n−1n
 MathType@MTEF@5@5@+=feaafiart1ev1aaatCvAUfKttLearuWrP9MDH5MBPbIqV92AaeXatLxBI9gBaebbnrfifHhDYfgasaacH8akY=wiFfYdH8Gipec8Eeeu0xXdbba9frFj0=OqFfea0dXdd9vqai=hGuQ8kuc9pgc9s8qqaq=dirpe0xb9q8qiLsFr0=vr0=vr0dc8meaabaqaciaacaGaaeqabaqabeGadaaakeaacqWGmbatcqWGtbWucqWGebarcqGH9aqpdaGcaaqaamaalaaabaGaeGOmaiJaeyOiGCRaemyta0Kaem4uamLaemyrau0aaSbaaSqaaiabdEha3jabdMgaPjabdsha0jabdIgaOjabdMgaPjabd6gaUbqabaGccqGHIaYTcqWGgbGrdaWgaaWcbaGaeGymaeJaeiilaWIaemOBa4MaeyOeI0IaeGymaedabeaaaOqaaiabd6gaUbaaaSqabaaaaa@48F3@

where the MSE_within _is from the above ANOVA calculation, the F-statistic is based on *k*-1 (in this case, equal to 1, because only two groups are being contrasted), *n *is the geometric mean of the *n*'s in the two groups being compared. The LSD then represents the minimum value of the difference between two means in order for their difference to be considered significant.

### Pattern ID

We combine results from all of the pairwise comparisons for each probe set, creating a pattern ID. Pattern IDs are constructed using logic gates that use 'increase', 'no significant change', or 'decrease' results from each pairwise comparison. The first pairwise comparison is assigned 1, 0, or -1; the second is assigned 10, 0, or -10; the third is assigned 100, 0, -100 and so on. In this way, the sum of each combination of pairwise comparisons for a given probe set creates a pattern ID encoding that pattern's statistically defined shape, and allowing researchers to easily group different genes that belong to the same pattern. Further, two patterns of opposite sign and the same absolute value will be mirror reflections of one another, which may have value for assessing opposing actions in single pathways [46].

### Z Score (probability of pattern's chance occurrence)

The distance, in standard deviations, of each pattern's prevalence in the real data compared to its prevalence in the Monte Carlo simulation is calculated using the Z-score as follows:

Z=(γ∑γ−R∑R)−0.5•(1∑γ+1∑R)(γ+R∑γ+∑R)•(1−γ+R∑γ+∑R)•(1∑γ+1∑R)
 MathType@MTEF@5@5@+=feaafiart1ev1aaatCvAUfKttLearuWrP9MDH5MBPbIqV92AaeXatLxBI9gBaebbnrfifHhDYfgasaacH8akY=wiFfYdH8Gipec8Eeeu0xXdbba9frFj0=OqFfea0dXdd9vqai=hGuQ8kuc9pgc9s8qqaq=dirpe0xb9q8qiLsFr0=vr0=vr0dc8meaabaqaciaacaGaaeqabaqabeGadaaakeaacqWGAbGwcqGH9aqpdaWcaaqaamaabmaabaWaaSaaaeaaiiGacqWFZoWzaeaadaaeabqaaiab=n7aNbWcbeqab0GaeyyeIuoaaaGccqGHsisldaWcaaqaaiabdkfasbqaamaaqaeabaGaemOuaifaleqabeqdcqGHris5aaaaaOGaayjkaiaawMcaaiabgkHiTiabicdaWiabc6caUiabiwda1iabgkci3oaabmaabaWaaSaaaeaacqaIXaqmaeaadaaeabqaaiab=n7aNbWcbeqab0GaeyyeIuoaaaGccqGHRaWkdaWcaaqaaiabigdaXaqaamaaqaeabaGaemOuaifaleqabeqdcqGHris5aaaaaOGaayjkaiaawMcaaaqaamaakaaabaWaaeWaaeaadaWcaaqaaiab=n7aNjabgUcaRiabdkfasbqaamaaqaeabaGae83SdCMaey4kaSYaaabqaeaacqWGsbGuaSqabeqaniabggHiLdaaleqabeqdcqGHris5aaaaaOGaayjkaiaawMcaaiabgkci3oaabmaabaGaeGymaeJaeyOeI0YaaSaaaeaacqWFZoWzcqGHRaWkcqWGsbGuaeaadaaeabqaaiab=n7aNjabgUcaRmaaqaeabaGaemOuaifaleqabeqdcqGHris5aaWcbeqab0GaeyyeIuoaaaaakiaawIcacaGLPaaacqGHIaYTdaqadaqaamaalaaabaGaeGymaedabaWaaabqaeaacqWFZoWzaSqabeqaniabggHiLdaaaOGaey4kaSYaaSaaaeaacqaIXaqmaeaadaaeabqaaiabdkfasbWcbeqab0GaeyyeIuoaaaaakiaawIcacaGLPaaaaSqabaaaaaaa@76FF@

where *γ *is the number of times a pattern appears in real data, Σ*γ *is the total for all unique patterns in real data, *R *is the number of times pattern appears in Monte Carlo, and Σ*R *is the total for all unique patterns in Monte Carlo.

### Software

#### Description

StatiGen is written in Visual Basic using the .NET 1.1 architecture and recapitulates all of the steps described above for the PPM algorithm. StatiGen also standardizes gene expression levels, allowing multiple genes in a single pattern to be averaged together and plotted. Graphic displays of this output are provided, along with lists of identified genes. StatiGen also creates hyperlinks for probe set ID information (these are relevant for Affymetrix-based data only). The program runs on Windows 2000/XP operating systems and its performance is generally improved by increased RAM. The installation file is Additional File 2 and source code is Additional File 3. Importantly, the code 'passes' information across Excel worksheets; therefore, Excel must be installed for the program to work. Up-to-date versions of the software will linked through our Departmental [74] and Microarray Core [75] websites.

#### User input

Users are required to provide signal intensity, presence absence call, and annotation data as either text files or Excel worksheets (the unique identifier- probe set ID- should always be in the same order in the leftmost column of all sheets, and the top row should contain title information), although StatiGen provides some protection from misaligned inputs by sorting by the first column and making sure the number of rows match across worksheet. A number of different signal intensity algorithms are available [76-78] (*e.g*., PLIER, MAS4, MAS5, RMA, gcRMA, DCHIP, GLA), and, within each of these, there are multiple settings. Therefore, StatiGen makes no assumptions regarding signal intensity transformation. Users should run their transformations (logging), etc. prior to running StatiGen. Presence absence calls (P, M, A) or p values (derived from Affymetrix-based algorithms), as well as user-defined spot quality flags, can be used to pre statistically-filter ('triage') data prior to statistical analysis. Because annotation is based on current knowledge and is therefore a 'moving target' [79], no attempt at annotation is provided with StatiGen. Instead, users provide their own annotation file. Importantly, this annotation file can have as many columns (up to the 255 column limit in Excel worksheets- less the leftmost unique ID column and the StatiGen generated hyperlink column) as the user would like.

### Original data

Data from one of our laboratory's earlier microarray studies [2] is provided to highlight StatiGen's functionality. Raw (.cel files), MAS4 signal intensity, and presence data are available through the Gene Expression Omnibus (GSE 854), and signal intensity (Additional File 4), presence call (Additional File 5) and annotation (Additional File 6) files are also provided with this manuscript. In that study, male Fischer 344 rats of three ages (3 month- Young, 12 month- Mid-Age, and 24 month- Aged; n = 9–10/group) were behaviorally characterized on two hippocampus-dependent cognitive tasks. Their hippocampi were removed, the CA1 regions dissected, and each animal's isolated RNA was hybridized to its own microarray (RG-U34A, Affymetrix; one chip per animal). Microarray analysis included a 1-Way ANOVA followed by *post hoc *Pearson's correlation between signal intensity and *pre mortem *behavioral scores.

## Authors' contributions

RH wrote StatiGen and associated help files, and EMB devised the algorithm and constructed prototype Excel files depicting its use.

## Supplementary Material

Additional file 1 Complete list of probe sets for the five identified patterns. Click here for file

Additional file 2 StatiGen installation file. Click here for file

Additional file 3 StatiGen source code. Click here for file

Additional file 4 Signal Intensity input file for StatiGen. Click here for file

Additional file 5 Presence call input file for StatiGen. Click here for file

Additional file 6 Annotation file for StatiGen. Click here for file
