# Supplementary material for: Post hoc pattern matching: assigning significance to statistically defined expression patterns in single channel microarray data
Source: BMC Bioinformatics. 2007 Jul 5;8:240. doi: 10.1186/1471-2105-8-240 (PMC1934919; doi:10.1186/1471-2105-8-240)
Supplement: Additional file 3 — StatiGen source code. [file 1471-2105-8-240-S3.zip › StatiGen_Source_06142007/bin/help/help1.htm]

Example overview topic


**Importing Data (STEP 1 of 6)**

---

This procedure will import raw data from your input files and begin to format it for use within the StatiGen
program.


1. **Project Name**  - Choose a descriptive
   name for your project.  This name will also serve a default file prefix
   for all StatiGen output files.

   - **Import Format** 
     - Your
     raw data must be in one of the following formats prior to import:
     - **Combined**
       - Signal intensities and
       presence call p-values (or P/A calls) are provided on a single sheet
       (see note on presence/absence
       call import formats).  The
       first column of the sheet must contain a list of probe set IDs.  See
       example below:
     - **Split** - Signal intensities are
       provided in one workbook, while presence calls are provided
       in a second
       workbook (see note on presence/absence
       call import formats).  On each sheet, the first column must contain a list of probe set
       IDs, preferably in the same order.
     - **Intensities Only**
       - A single workbook
       containing signal intensities is provided (without presence calls).  The
       first column must contain a list of probe set IDs.  The format is exactly the same as for the signal intensities file
       shown in the split format above.- **Input File(s)** -Select your input file or files by clicking the browse button and navigating to
       the corresponding files.

       - **Annotation File** -You must provide annotation on a single sheet, with the first column containing
         a list of probe set IDs.  The second column should contain a list of gene
         symbols and some text marker (e.g. 'EST', '---', etc.) that
         is repeated for those probesets with no gene symbol
         level annotation .  You will have the opportunity
         to filter based on this marker at a later time.  You may also provide custom annotation fields (columns 3 through
         9) containing any information you wish.

         - Note - The first row should contain column titles.


1. **Import** - When you have completely
   filled out the form, click the 'Import' button to continue.

|  |
| --- |
| **IMPORTANT NOTE ON PRESENCE/ABSENCE CALL IMPORT FORMATS**  Presence/absence calls can be provided as p-values (numerical values from 0 to 1) or as text ('P' = present; 'M' = marginal; 'A' = absent).  StatiGen automatically detects the format of presence/absence calls and adjusts downstream analysis accordingly.  For presence call p-values, users can adjust the p-value threshold at which probesets are declared present.  For "P/M/A" text, 'P's and 'M's are counted as present and 'A's are counted as absent. |
