# Supplementary material for: Post hoc pattern matching: assigning significance to statistically defined expression patterns in single channel microarray data
Source: BMC Bioinformatics. 2007 Jul 5;8:240. doi: 10.1186/1471-2105-8-240 (PMC1934919; doi:10.1186/1471-2105-8-240)
Supplement: Additional file 3 — StatiGen source code. [file 1471-2105-8-240-S3.zip › StatiGen_Source_06142007/bin/help/help2.htm]

Example overview topic


**Setting
Up Experimental Groups (STEP 2 of 6)**

---

In this step, you will set up
experimental groups by creating a group name list, adding chips to groups and
then confirming your group setup.  These groups will then be used for
subsequent analyses.


**General
Procedure:**

1. **Add Group Name
   to the Groups List**
   - Type a group name into the group
     name field (located under the 'Groups' box).

     - Click the 'New' button to add it
       to the 'Groups' list.  The group name will now appear in the 'Groups'
       list.

       - Repeat the procedure to add
         additional groups to the list.

         - **Note**:  You must add **at
           least 2 groups** to the list.- **Add Chips to
     Your Groups** (see "Other
     Procedures" for editing groups and group membership)
     - Select a group from the 'Groups
       List' by clicking on its name.

       - Select chips you wish to add to
         the group from the 'Available Chips' list by clicking on each chip name. 
         A checkmark will appear in the box to the left
         of the selected chip name.

         - Click the 'Add Chips to Group'
           button.

           - The chip names will now appear in
             the 'Chips in Group' list at the bottom left of the window.

             - Repeat the procedure for each
               group you have created.

               - When you have finished adding
                 chips to groups, click the 'Next' button at the bottom right of the window to
                 advance to the confirmation window.


1. **Confirm Group
   Setup**
   - Review all groups and be sure they
     are correct.

     - If groups are correct, click the
       'Next' button.  You have successfully created your experimental groups.

******---****Other
Procedures:**


1. **Remove Chip from
   Selected Group**
   - Select group from 'Groups List' by
     clicking its name.

     - Select the name of the chip you
       wish to remove from the group by clicking its name in the 'Chips in Group'
       list.

       - Click the 'Remove' button. 
         The chip will be returned to the 'Available Chips' list and will be visible at
         the bottom of the list.- **Delete Group
     from the 'Groups List'**
     - Select the group you wish to
       delete by clicking on its name in the 'Groups List'.

       - Click the 'Delete' button. 
         The group name will be deleted and all chips (if any) contained in the group
         will be returned to the 'Available Chips' list and will be visible at the
         bottom of the list.
