# Supplementary material for: Post hoc pattern matching: assigning significance to statistically defined expression patterns in single channel microarray data
Source: BMC Bioinformatics. 2007 Jul 5;8:240. doi: 10.1186/1471-2105-8-240 (PMC1934919; doi:10.1186/1471-2105-8-240)
Supplement: Additional file 3 — StatiGen source code. [file 1471-2105-8-240-S3.zip › StatiGen_Source_06142007/bin/help/help3.htm]

Example overview topic


**Creating the Master Data File (STEP 3 of 6)**

---

This procedure will create a
master data file (.xls format) in your project folder which will contain
StatiGen formatted signal intensity, presence call, and annotation data as well
as an unfiltered pattern summary for all data.


1. **Project
   Destination Folder**
   - Type or select (click 'browse') a
     valid path to your project folder.

     - The default folder is set to the
       folder where you installed StatiGen in a subfolder called 'output':
       - **Example**:  c:\Program
         Files\UKCOM\StatiGen\output\- **Note**:  All files
         created by StatiGen for your project will be placed into this folder.- **Master File Name**
     - This will be the name of your
       'Master Data File'.

       - By default, all files names use
         the dot notation scheme as follows:
         - Dot Notation Scheme: 
           'ProjectName'.'Output'.'Extension'

           - **Example**: If your project was called
             'MyProject' and StatiGen is building the 'Master Data File', the default
             filename is:  MyProject.master.xls

             - You may choose to change the
               filename or leave it as the default.- **ANOVA P-Value**
       - This value is used as a cutoff for
         the ANOVA test performed on your data for each probeset ID prior to assigning a
         pattern code to any pairwise comparison within that data.

         - For genes/probesets in which the ANOVA value is higher than the
           criterion value you specify in this box, all pairwise comparison tests
           for that probeset ID will receive a '0', and the overall pattern
           will equal '0' for that probeset ID.
           - **Note**:  All probeset
             IDs with '0' overall patterns are by definition not statistically significant
             and will not be included in further analyses.- **Presence/Absence
         Call P-Value**
         - This value is used during the
           determination of Presence/Absence calls based on P-value data you have provided
           to StatiGen.
           - **Note**:  If you have
             not supplied P/A data, or if you have supplied P/A data as text values(e.g.
             'P'; 'M'; 'A'), then this option will not be available.- Only data with
             corresponding P-Values equal to or lower than the value you specify in this box
             will be considered "Present".
             - **Note**:  This setting
               should not be confused with 'P-Value' cutoff, which is available during the
               Filter stage (this allows you to set a limit on data based on
               # P/A calls in the dataset).- **When you have
           finished filling in the form, click 'Next' to continue.**
