# Supplementary material for: Post hoc pattern matching: assigning significance to statistically defined expression patterns in single channel microarray data
Source: BMC Bioinformatics. 2007 Jul 5;8:240. doi: 10.1186/1471-2105-8-240 (PMC1934919; doi:10.1186/1471-2105-8-240)
Supplement: Additional file 3 — StatiGen source code. [file 1471-2105-8-240-S3.zip › StatiGen_Source_06142007/bin/help/help4.htm]

Example overview topic


**Creating the Filtered Data File (STEP 4 of 6)**

---

This procedure will create a
filtered data file (.xls format) in your project folder which will contain
filtered StatiGen formatted signal intensity, presence call, and annotation
data, as well as a filtered pattern list, significant pattern summary, and a
significant gene list.


1. **Filtered File
   Name**
   - This will be the name of your
     'Filtered Data File'.

     - By default, all files names use
       the dot notation scheme as follows:
       - Dot Notation Scheme: 
         'ProjectName'.'Output'.'Extension'

         - **Example**: If your project was called
           'MyProject' and StatiGen is building the 'Filtered Data File', the default
           filename is:  MyProject.filtered.xls

           - You may choose to change the
             filename or leave it as the default.- **Presence Call
     Cutoff**
     - This value is used as a cutoff
       based on the number of chips with 'Present' data
       for a particular probeset/gene as determined by the 'Presence
       Call P-Value' in the previous step.

       - Only those probeset IDs with an
         equal or greater number of 'Present'
         calls will be included in the
         filtered output.- **Annotation****Flag**
       - You may choose to specify a custom
         flag to indicate unannotated probesets in your annotation data.
         - This flag should be present in the
           'Gene Symbol' (second) column of the annotation file you supplied to StatiGen.

           - You may only specify a single flag.- This flag will be used to filter
           out data associated with probeset IDs that are flagged as
           unannotated.  These
           will not be included in the filtered output.- **Include
         Comparisons**
         - Use this checkbox list to select
           which pairwise comparisons you wish to include in the filtered data (and
           therefore in the final analysis).
           - **Note**: 
             You must select at least 1
             pairwise comparison.- You may select all comparisons by
             clicking 'Select All', or unselect all comparisons by clicking 'Unselect All'.- **When you have
           finished filling in the form, click 'Next' to continue.**
