# Supplementary material for: Post hoc pattern matching: assigning significance to statistically defined expression patterns in single channel microarray data
Source: BMC Bioinformatics. 2007 Jul 5;8:240. doi: 10.1186/1471-2105-8-240 (PMC1934919; doi:10.1186/1471-2105-8-240)
Supplement: Additional file 3 — StatiGen source code. [file 1471-2105-8-240-S3.zip › StatiGen_Source_06142007/bin/help/help5.htm]

Example overview topic


**Creating the Final Report (STEP 5 of 6)**

---

This procedure will create a gene
list (in your project folder) grouped into patterns that have been calculated
to have statistical significance within your data.  A MonteCarlo
simulation is used to test your actual data against randomly generated
data.  This gene list will be viewable using the 'StatiGen Viewer' 
after analysis is complete.  This file will include a graphical explorer
to help you view your significant patterns and export gene lists for further
analysis.


1. **Final Gene List
   File Name**
   - This will be the name of your
     'Final Gene List Data File'.

     - By default, all files names use
       the dot notation scheme as follows:
       - Dot Notation Scheme: 
         'ProjectName'.'Output'.'Extension'

         - **Example**: If your project was called
           'MyProject' and StatiGen is building the 'Final Gene List Data File', the
           default filename is:  MyProject.viewer.xls

           - You may choose to change the
             filename or leave it as the default.- **MonteCarlo
     Simulation**
     - Select the number of times you
       wish the MonteCarlo to be repeated.
       - **Note**: 
         Choosing too few iterations can lead
         to unstable results, while choosing
         too many will waste time.  The
         number of iterations at which an
         analysis becomes unstable is
         dependent on the number of groups in
         the study.  To test for
         stability, re-run the analysis with
         the same number of iterations. 
         If the pattern order is the same and
         the significance is similar, then
         that number of iterations is
         probably appropriate.  If the
         results are not stable, then
         increase the number of iterations
         (e.g. double them) and try again.- **Z-Score Cutoff**
       - Significant patterns will be determined by a
         Z-test (shown below).

> - Z-scores will be used as cutoffs in the determination of significant patterns.  Only those patterns
>   with a Z-Score higher than a user-selected
>   value (default = 2) will be included in the 'Final Gene List'.
> - For a list of all patterns, the Z-score
>   filter can be disabled via a clickbox.

1. **When you have
   finished filling in the form, click 'Next' to continue.**
