# Supplementary material for: Post hoc pattern matching: assigning significance to statistically defined expression patterns in single channel microarray data
Source: BMC Bioinformatics. 2007 Jul 5;8:240. doi: 10.1186/1471-2105-8-240 (PMC1934919; doi:10.1186/1471-2105-8-240)
Supplement: Additional file 3 — StatiGen source code. [file 1471-2105-8-240-S3.zip › StatiGen_Source_06142007/bin/help/help6.htm]

Example overview topic


**Using the StatiGen Viewer (STEP 6 of 6)**

---

When your project is complete, the
following window will be displayed.  To open the StatiGen Viewer, click
the View button, or on the filename listed under "Final Gene List Data
File".  You may also open the filtered and master data files by clicking
their respective filenames.


The StatiGen Viewer is an Excel
file that includes your final gene list.  The graphical explorer will
allow you to view important statistics and a chart of describing each
significant pattern.  You may then choose to export gene lists associated
with specific patterns to another Excel file.

- **Study Summary**
  - Project Name

    - Number of statistically
      significant patterns and the total number of genes contained in those patterns.- **Pattern Summary**
    - Pattern ID:  The numerical
      code assigned to this unique pattern.

      - Rank:  This field indicates
        the relative significance of this pattern as compared to the other
        statistically significant patterns found.

        - Z-Score:  The distance (in standard deviations)
          of this pattern's prevalence in the actual data
          compared to its prevalence in the random data.

          - P-Value:  The probability
            that such a pattern could contain that number of
            genes by chance (based on Z-score).

            - # Genes:  The number of genes
              matching this pattern.- **Pattern Selector**
      - Click this drop down list and
        select the desired pattern to view.

        - Includes the Pattern ID and
          relative rank value.- **Graphical
        Pattern Viewer**
        - Displays the pattern graphically.

          - Each gene within a pattern has its standardized mean
            calculated per treatment group.  The treatment
            group means for different genes are then averaged
            together and plotted as mean +/- SEM based on the
            number of genes in the pattern.- **Pattern Export**
          - Click this button to export the
            currently viewed pattern gene list to an Excel file.- **Gene List**
            - This list contains all genes
              matching the selected pattern.

              - Includes the Probe Set ID, Gene
                Symbol, Gene Name, and any optional fields you supplied with your annotation
                file.
