# Supplementary figures and images for: Post hoc pattern matching: assigning significance to statistically defined expression patterns in single channel microarray data
Source: BMC Bioinformatics. 2007 Jul 5;8:240. doi: 10.1186/1471-2105-8-240 (PMC1934919; doi:10.1186/1471-2105-8-240)

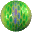

Supplement: Additional file 3 — StatiGen source code. [file 1471-2105-8-240-S3.zip › StatiGen_Source_06142007/bin/help/images/1or001a.gif]

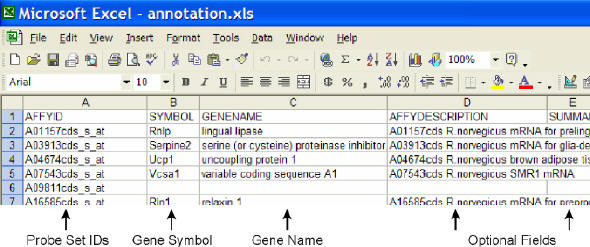

Supplement: Additional file 3 — StatiGen source code. [file 1471-2105-8-240-S3.zip › StatiGen_Source_06142007/bin/help/images/annotation.JPG]

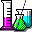

Supplement: Additional file 3 — StatiGen source code. [file 1471-2105-8-240-S3.zip › StatiGen_Source_06142007/bin/help/images/Chem.gif]

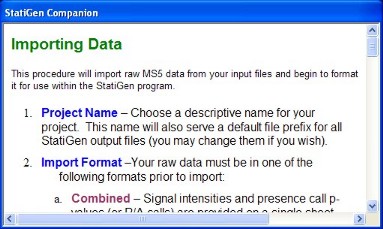

Supplement: Additional file 3 — StatiGen source code. [file 1471-2105-8-240-S3.zip › StatiGen_Source_06142007/bin/help/images/companion.jpg]

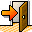

Supplement: Additional file 3 — StatiGen source code. [file 1471-2105-8-240-S3.zip › StatiGen_Source_06142007/bin/help/images/door02.gif]

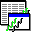

Supplement: Additional file 3 — StatiGen source code. [file 1471-2105-8-240-S3.zip › StatiGen_Source_06142007/bin/help/images/filtered-data.png]

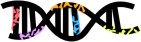

Supplement: Additional file 3 — StatiGen source code. [file 1471-2105-8-240-S3.zip › StatiGen_Source_06142007/bin/help/images/gene.jpg]

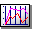

Supplement: Additional file 3 — StatiGen source code. [file 1471-2105-8-240-S3.zip › StatiGen_Source_06142007/bin/help/images/graph05.gif]

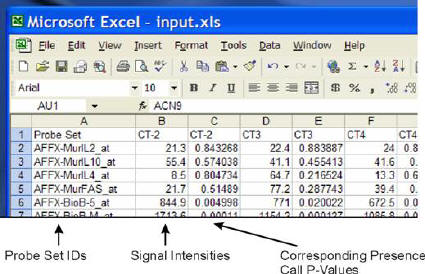

Supplement: Additional file 3 — StatiGen source code. [file 1471-2105-8-240-S3.zip › StatiGen_Source_06142007/bin/help/images/image001.jpg]

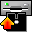

Supplement: Additional file 3 — StatiGen source code. [file 1471-2105-8-240-S3.zip › StatiGen_Source_06142007/bin/help/images/install.gif]

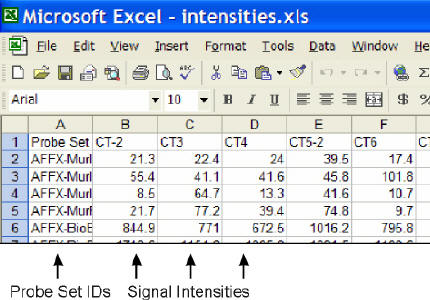

Supplement: Additional file 3 — StatiGen source code. [file 1471-2105-8-240-S3.zip › StatiGen_Source_06142007/bin/help/images/intensitiesonly.JPG]

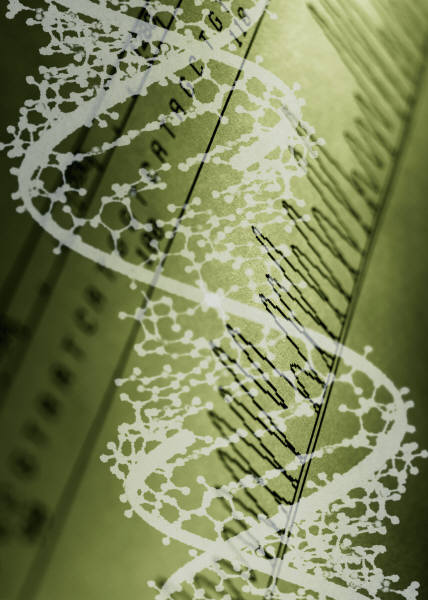

Supplement: Additional file 3 — StatiGen source code. [file 1471-2105-8-240-S3.zip › StatiGen_Source_06142007/bin/help/images/introducing.jpg]

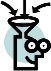

Supplement: Additional file 3 — StatiGen source code. [file 1471-2105-8-240-S3.zip › StatiGen_Source_06142007/bin/help/images/know.jpg]

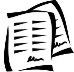

Supplement: Additional file 3 — StatiGen source code. [file 1471-2105-8-240-S3.zip › StatiGen_Source_06142007/bin/help/images/license.jpg]

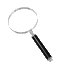

Supplement: Additional file 3 — StatiGen source code. [file 1471-2105-8-240-S3.zip › StatiGen_Source_06142007/bin/help/images/m-glass-a.gif]

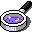

Supplement: Additional file 3 — StatiGen source code. [file 1471-2105-8-240-S3.zip › StatiGen_Source_06142007/bin/help/images/Magnify0b.gif]

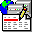

Supplement: Additional file 3 — StatiGen source code. [file 1471-2105-8-240-S3.zip › StatiGen_Source_06142007/bin/help/images/master-data.png]

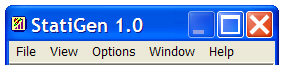

Supplement: Additional file 3 — StatiGen source code. [file 1471-2105-8-240-S3.zip › StatiGen_Source_06142007/bin/help/images/menu.jpg]

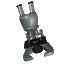

Supplement: Additional file 3 — StatiGen source code. [file 1471-2105-8-240-S3.zip › StatiGen_Source_06142007/bin/help/images/microscope-a.gif]

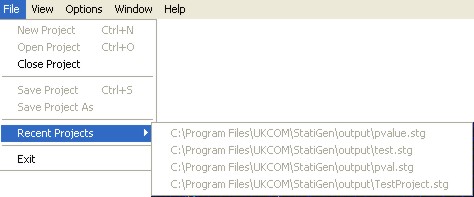

Supplement: Additional file 3 — StatiGen source code. [file 1471-2105-8-240-S3.zip › StatiGen_Source_06142007/bin/help/images/mnu_File.jpg]

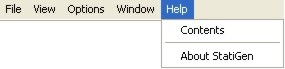

Supplement: Additional file 3 — StatiGen source code. [file 1471-2105-8-240-S3.zip › StatiGen_Source_06142007/bin/help/images/mnu_Help.jpg]

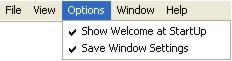

Supplement: Additional file 3 — StatiGen source code. [file 1471-2105-8-240-S3.zip › StatiGen_Source_06142007/bin/help/images/mnu_Options.jpg]

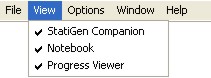

Supplement: Additional file 3 — StatiGen source code. [file 1471-2105-8-240-S3.zip › StatiGen_Source_06142007/bin/help/images/mnu_View.jpg]

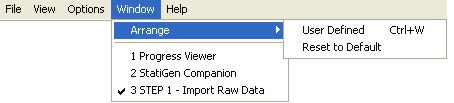

Supplement: Additional file 3 — StatiGen source code. [file 1471-2105-8-240-S3.zip › StatiGen_Source_06142007/bin/help/images/mnu_Window.jpg]

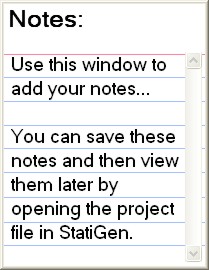

Supplement: Additional file 3 — StatiGen source code. [file 1471-2105-8-240-S3.zip › StatiGen_Source_06142007/bin/help/images/notes.jpg]

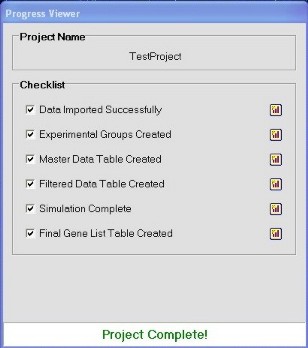

Supplement: Additional file 3 — StatiGen source code. [file 1471-2105-8-240-S3.zip › StatiGen_Source_06142007/bin/help/images/progressviewer.jpg]

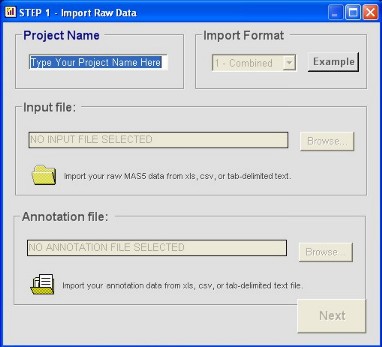

Supplement: Additional file 3 — StatiGen source code. [file 1471-2105-8-240-S3.zip › StatiGen_Source_06142007/bin/help/images/projectwindow.jpg]

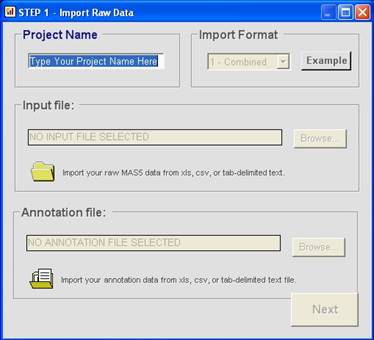

Supplement: Additional file 3 — StatiGen source code. [file 1471-2105-8-240-S3.zip › StatiGen_Source_06142007/bin/help/images/pw.jpg]

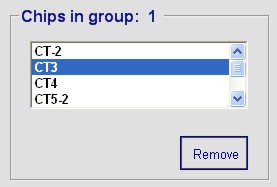

Supplement: Additional file 3 — StatiGen source code. [file 1471-2105-8-240-S3.zip › StatiGen_Source_06142007/bin/help/images/removechips.jpg]

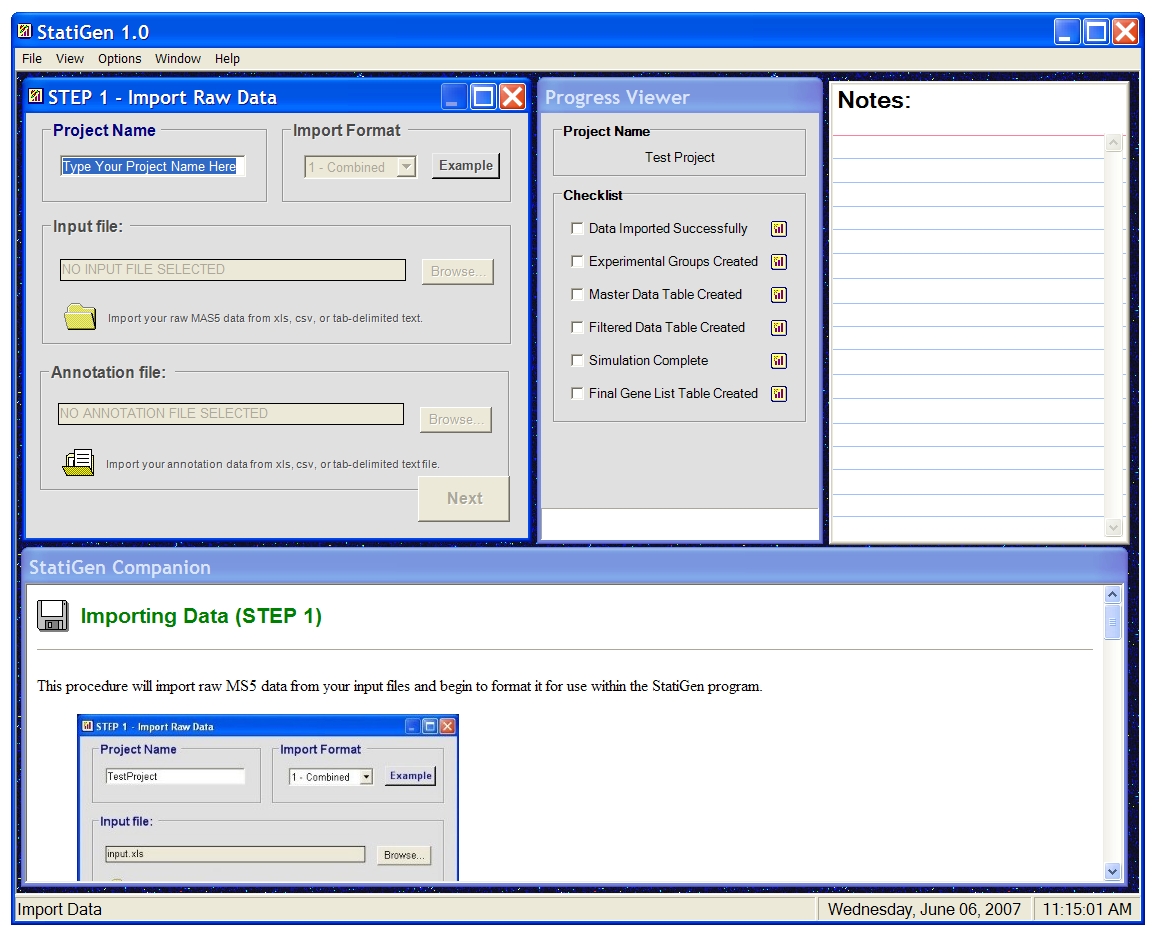

Supplement: Additional file 3 — StatiGen source code. [file 1471-2105-8-240-S3.zip › StatiGen_Source_06142007/bin/help/images/shebang.jpg]

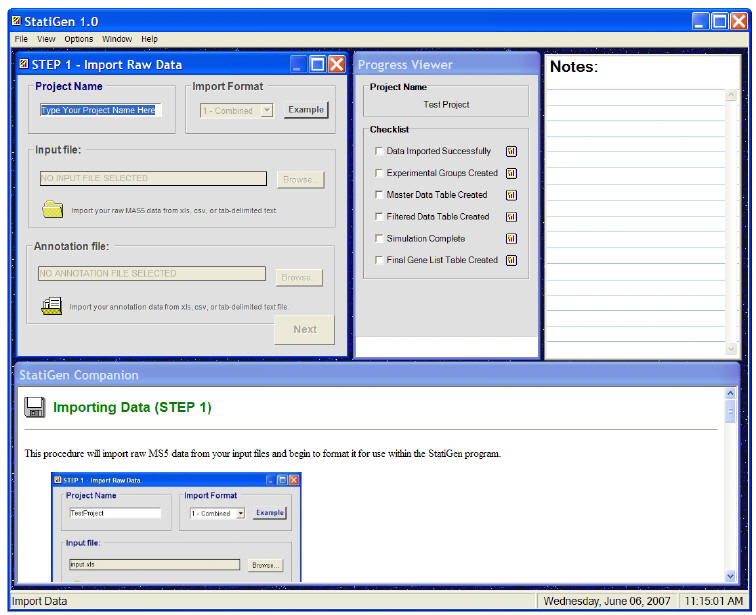

Supplement: Additional file 3 — StatiGen source code. [file 1471-2105-8-240-S3.zip › StatiGen_Source_06142007/bin/help/images/shebang_s.jpg]

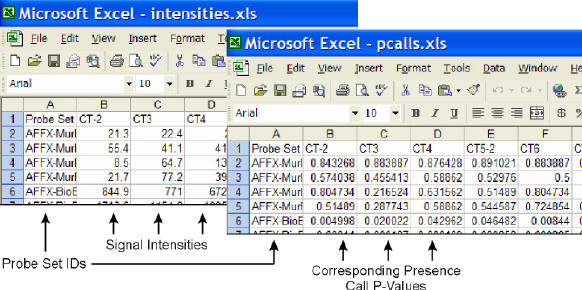

Supplement: Additional file 3 — StatiGen source code. [file 1471-2105-8-240-S3.zip › StatiGen_Source_06142007/bin/help/images/split.JPG]

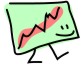

Supplement: Additional file 3 — StatiGen source code. [file 1471-2105-8-240-S3.zip › StatiGen_Source_06142007/bin/help/images/statigen.jpg]

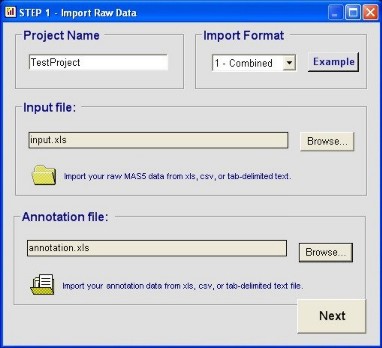

Supplement: Additional file 3 — StatiGen source code. [file 1471-2105-8-240-S3.zip › StatiGen_Source_06142007/bin/help/images/step1.jpg]

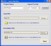

Supplement: Additional file 3 — StatiGen source code. [file 1471-2105-8-240-S3.zip › StatiGen_Source_06142007/bin/help/images/step1s.jpg]

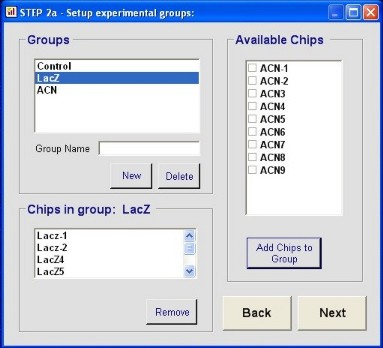

Supplement: Additional file 3 — StatiGen source code. [file 1471-2105-8-240-S3.zip › StatiGen_Source_06142007/bin/help/images/step2a.jpg]

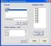

Supplement: Additional file 3 — StatiGen source code. [file 1471-2105-8-240-S3.zip › StatiGen_Source_06142007/bin/help/images/step2as.jpg]

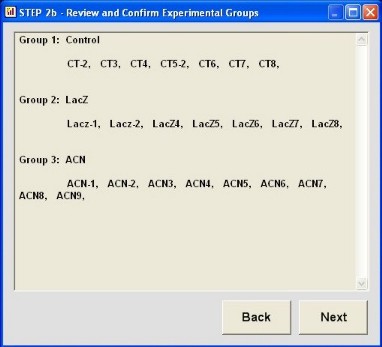

Supplement: Additional file 3 — StatiGen source code. [file 1471-2105-8-240-S3.zip › StatiGen_Source_06142007/bin/help/images/step2b.jpg]

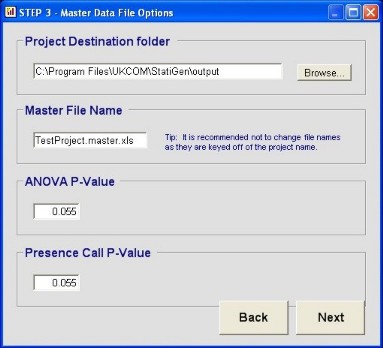

Supplement: Additional file 3 — StatiGen source code. [file 1471-2105-8-240-S3.zip › StatiGen_Source_06142007/bin/help/images/step3.jpg]

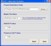

Supplement: Additional file 3 — StatiGen source code. [file 1471-2105-8-240-S3.zip › StatiGen_Source_06142007/bin/help/images/step3s.jpg]

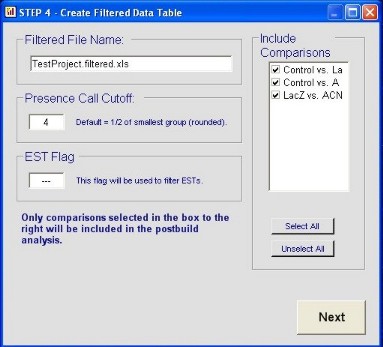

Supplement: Additional file 3 — StatiGen source code. [file 1471-2105-8-240-S3.zip › StatiGen_Source_06142007/bin/help/images/step4.jpg]

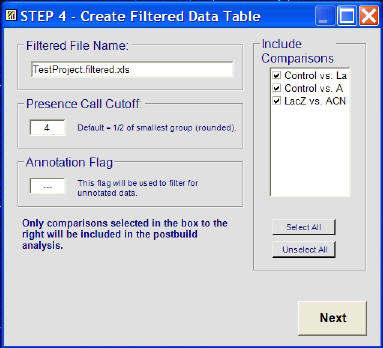

Supplement: Additional file 3 — StatiGen source code. [file 1471-2105-8-240-S3.zip › StatiGen_Source_06142007/bin/help/images/step4new.jpg]

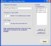

Supplement: Additional file 3 — StatiGen source code. [file 1471-2105-8-240-S3.zip › StatiGen_Source_06142007/bin/help/images/step4s.jpg]

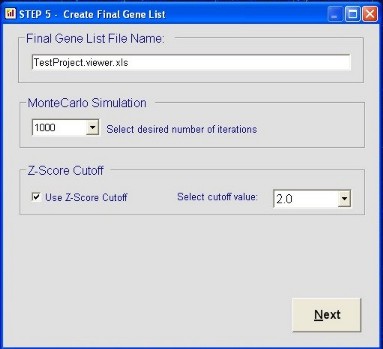

Supplement: Additional file 3 — StatiGen source code. [file 1471-2105-8-240-S3.zip › StatiGen_Source_06142007/bin/help/images/step5.jpg]

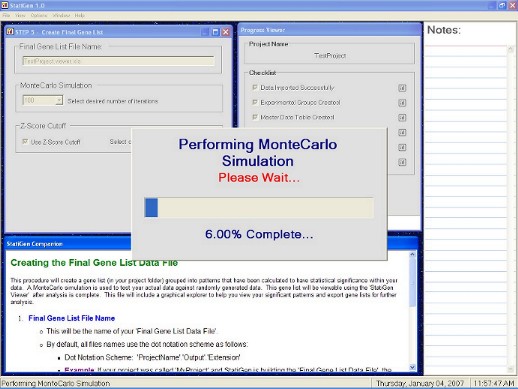

Supplement: Additional file 3 — StatiGen source code. [file 1471-2105-8-240-S3.zip › StatiGen_Source_06142007/bin/help/images/step5mc.jpg]

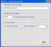

Supplement: Additional file 3 — StatiGen source code. [file 1471-2105-8-240-S3.zip › StatiGen_Source_06142007/bin/help/images/step5s.jpg]

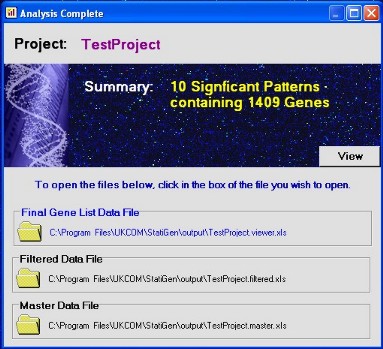

Supplement: Additional file 3 — StatiGen source code. [file 1471-2105-8-240-S3.zip › StatiGen_Source_06142007/bin/help/images/step6.jpg]

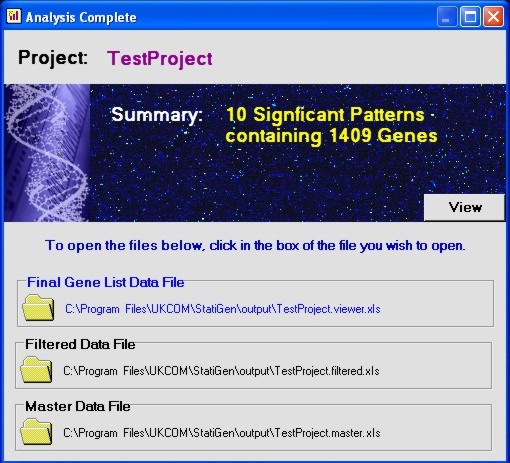

Supplement: Additional file 3 — StatiGen source code. [file 1471-2105-8-240-S3.zip › StatiGen_Source_06142007/bin/help/images/step6complete.jpg]

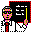

Supplement: Additional file 3 — StatiGen source code. [file 1471-2105-8-240-S3.zip › StatiGen_Source_06142007/bin/help/images/Teacher.gif]

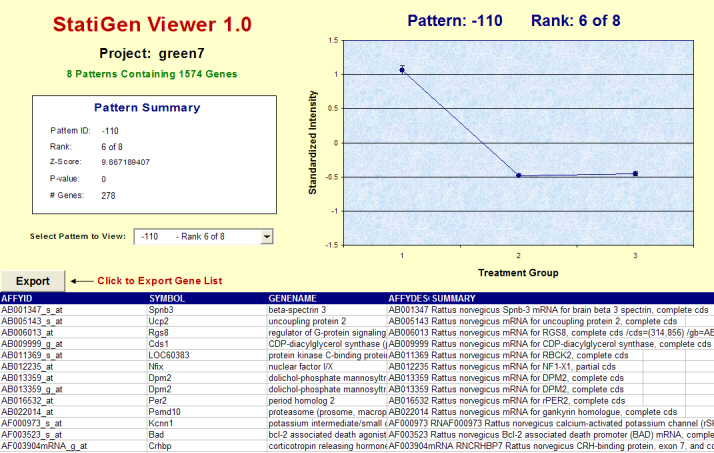

Supplement: Additional file 3 — StatiGen source code. [file 1471-2105-8-240-S3.zip › StatiGen_Source_06142007/bin/help/images/viewer.jpg]

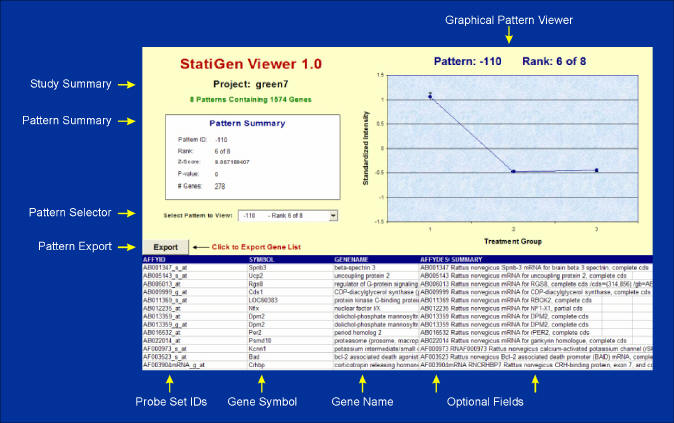

Supplement: Additional file 3 — StatiGen source code. [file 1471-2105-8-240-S3.zip › StatiGen_Source_06142007/bin/help/images/viewerpic.jpg]

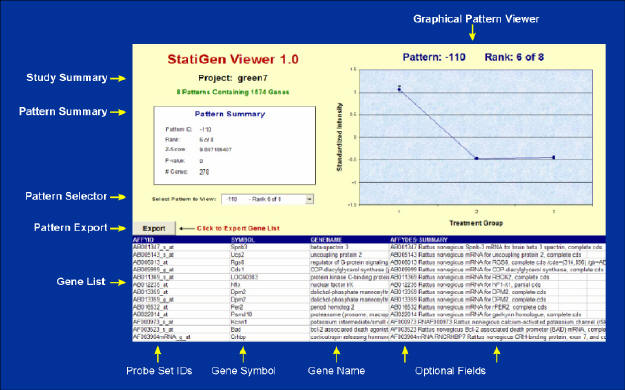

Supplement: Additional file 3 — StatiGen source code. [file 1471-2105-8-240-S3.zip › StatiGen_Source_06142007/bin/help/images/viewerpic2.jpg]

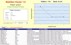

Supplement: Additional file 3 — StatiGen source code. [file 1471-2105-8-240-S3.zip › StatiGen_Source_06142007/bin/help/images/viewers.jpg]

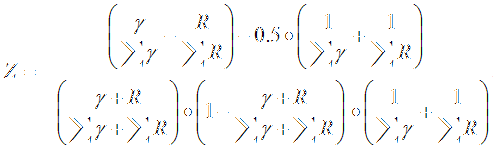

Supplement: Additional file 3 — StatiGen source code. [file 1471-2105-8-240-S3.zip › StatiGen_Source_06142007/bin/help/images/zscore.gif]

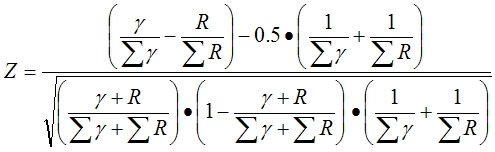

Supplement: Additional file 3 — StatiGen source code. [file 1471-2105-8-240-S3.zip › StatiGen_Source_06142007/bin/help/images/zscore.jpg]
